# Distribution of α-synuclein in the spinal cord and dorsal root ganglia in an autopsy cohort of elderly persons

**DOI:** 10.1186/s40478-015-0236-9

**Published:** 2015-09-15

**Authors:** Hiroyuki Sumikura, Masaki Takao, Hiroyuki Hatsuta, Shinji Ito, Yuta Nakano, Akiko Uchino, Akane Nogami, Yuko Saito, Hideki Mochizuki, Shigeo Murayama

**Affiliations:** Department of Neuropathology (the Brain Bank for Aging Research), Tokyo Metropolitan Geriatric Hospital & Institute of Gerontology, 35-2 Sakae-cho, Itabashi-ku, Tokyo 173-0015 Japan; Department of Neurology, Osaka University Graduate School of Medicine, Suita-shi, Osaka Japan; Department of Neurology, Saitama Medical University International Medical Center, Hidaka-shi, Saitama Japan; Department of Laboratory Medicine, National Center of Neurology and Psychiatry, Kodaira-shi, Tokyo Japan

## Abstract

**Background:**

Lewy body–related α-synucleinopathy (LBAS, the abnormal accumulation of pathologic α-synuclein) is found in the central and peripheral nervous systems, including the spinal cord, dorsal root ganglia, and sympathetic ganglia, of Parkinson’s disease patients. However, few studies have focused on the distribution of LBAS in the spinal cord, primary sensory neurons, and preganglionic sympathetic nerves.

**Results:**

We analyzed 265 consecutive subjects with LBAS who underwent autopsy at a general geriatric hospital. LBAS in the spinal cord was significantly associated with that in the lower brainstem regions that are directly connected to the spinal cord (i.e., the medullary reticular formation and locus ceruleus), but it was not associated with the olfactory bulb–amygdala system, which is not directly connected to the spinal cord, suggesting that the lower brainstem is a key structure regarding the spread of LBAS to the spinal cord. In the primary sensory neurons, most subjects with LBAS in the dorsal root ganglia had LBAS in the dorsal root, and all subjects with LBAS in the dorsal root also had LBAS in the dorsal horn, suggesting that LBAS spreads retrogradely from the axonal terminals of the dorsal horn to the somata of the dorsal root ganglia via the dorsal root. In the preganglionic sympathetic nerves, the LBAS in the sympathetic ganglia preceded that in the nucleus of the intermediolateral column of the thoracic cord, suggesting that LBAS spreads retrogradely through the preganglionic sympathetic nerves.

**Conclusions:**

LBAS in the spinal cord was associated with the lower regions of the brainstem, but not with the olfactory bulb or amygdala. LBAS may spread centrifugally along the primary sensory neurons, whereas it may spread centripetally along the preganglionic sympathetic nerves.

**Electronic supplementary material:**

The online version of this article (doi:10.1186/s40478-015-0236-9) contains supplementary material, which is available to authorized users.

## Background

Lewy bodies, neurites and dots are aggregates composed mainly of α-synuclein and are pathological hallmarks of Lewy body diseases, which include Parkinson’s disease and dementia with Lewy bodies [1]. In patients with Lewy body disease, these aggregates are found not only in the brain, but also in the spinal cord, sympathetic ganglia, and cardiac plexuses [2–4]. Recent studies have shown that pathologic α-synuclein may propagate from one cell to another and spread to different interconnected brain regions [5–15]. Lewy body–related α-synucleinopathy (LBAS, the abnormal accumulation of pathologic α-synuclein) in the brain spreads in a sequential, predictable fashion, that is, it spreads rostrally from the brainstem in a process described by the Braak hypothesis [16, 17]. In addition to this brainstem-ascending pathway, an alternative pathway, the olfactory bulb–amygdala pathway, has been reported to be present in up to 30 % of cases of Lewy body disease [18, 19].

LBAS in the spinal cord may begin to develop in specific regions such as the dorsal horn, intermediolateral column of the thoracic cord (Th-IML), and intermediolateral column of the sacral cord (Sa-IML) [20]. So far, studies investigating LBAS in the spinal cord have only examined subjects in which the spread of LBAS was following the brainstem-ascending pathway of LBAS and not the olfactory bulb–amygdala pathway [20–22].

Anatomical studies have revealed that, unlike the olfactory bulb and the amygdala, the lower brainstem nuclei, including the medullary reticular formation, locus ceruleus, and raphe nucleus, are directly connected to the spinal gray matter via projecting neurons [23–26]. Little is known about whether the spread of LBAS to the spinal cord is different in patients in whom the LBAS is spreading following the brainstem-ascending pathway compared with those in which it is spreading following the olfactory bulb–amygdala pathway. Similarly, the distribution of LBAS in the primary sensory neurons, whose cell bodies are located within the dorsal root ganglia (DRG) and axons extend into the dorsal horn via the dorsal root, is yet to be fully examined.

The sympathetic ganglia, which are innervated by the nucleus of the Th-IML, are known to be where LBAS manifests in the early phase of the disease process [3]. However the association between LBAS in the sympathetic ganglia and that in the Th-IML is yet to be fully elucidated.

According to the hypothesis described by Braak and colleagues [16], the dorsal motor nucleus of the vagus is one of the first regions in which LBAS develops, suggesting that the pathological process of Lewy body diseases may begin in the gut. The preganglionic parasympathetic nerves that innervate the gut are comprised of axons whose cell bodies are located within the nucleus of the Sa-IML; however, it is not yet fully understood whether the Sa-IML is also one of the primary sites in the spinal cord where LBAS begins to develop.

In the present study, we investigated the distribution of LBAS in the spinal cord, primary sensory neurons, and preganglionic sympathetic nerves in a consecutive autopsy series of elderly subjects.

## Materials and methods

### Subject selection

Samples of brain and spinal cord were collected from 796 consecutive full autopsies of elderly persons conducted in our institution between January 2003 and July 2013. Paraffin sections of the lumbosacral cord, thoracic cord, medulla, pons, midbrain, olfactory bulb, amygdala, hippocampus, cervicothoracic level of the sympathetic ganglia, heart, esophagus, and brachial skin were prepared and subjected to immunohistochemical analyses. Of the 796 subjects examined, 265 had LBAS in at least one of the anatomical regions examined.

Next, paraffin sections of the cervical cord, lumbosacral level of the DRG, and sites stipulated in the third report of the Dementia with Lewy Bodies Consortium [27] were analyzed as described below. C8, Th8, L2, L5, S1, and S2 were evaluated as representative levels of the spinal cord.

Neuropathological diagnoses were based on the third report of the Dementia with Lewy Bodies Consortium [27], our institute’s original criteria [28], Braak staging of β-amyloid and neurofibrillary tangle formation [29], Consortium to Establish a Registry for Alzheimer’s Disease [30], Thal phase evaluation [31], and argyrophilic grain staging [32].

The relatives’ informed consent was obtained from all subjects. Our Brain Bank for Aging Research was approved by the ethics committee of Tokyo Metropolitan Geriatric Hospital (No. 16).

The following aspects were analyzed in this study:

(A) Presence of LBAS in the spinal cord versus that in the brain (*n* = 265).
(B) Presence of LBAS in the spinal cord versus that in the (both *n* = 265).
(C) Presence of LBAS in the S2 dorsal horn versus that in specific regions of the brainstem (medullary reticular formation or locus ceruleus; *n* = 261, 260, respectively) or that in the amygdala (*n* = 261). The S2 dorsal horn was selected as the representative site of the spinal cord because lower level of spinal cord usually contains the greatest amount of LBAS deposits [3, 20].
(D) Distribution of LBAS in the primary sensory neurons at the S2, S1, L5, and L2 levels (*n* = 261, 46, 261, and 225, respectively).
(E) Presence of LBAS in the Th-IML versus that in the sympathetic ganglia (*n* = 222).
(F) Comparison of LBAS density in four different regions of the spinal cord: dorsal horn (Rexed lamina I–VI, *n* = 256), ventral horn (Rexed lamina VIII–IX, *n* = 258), intermediate zone (Rexed lamina VII and X, *n* = 258), and large motor neurons in the ventral horn (*n* = 255). Identification of large motor neurons was based on morphological features, including large, multipolar cell bodies containing large Nissl bodies.
(G) Confirmation of the presence of LBAS in the Sa-IML (*n* = 261).

### Histology

At the time of autopsy, half of the brain was preserved at −80 °C, and the other half was fixed in 20 % neutral-buffered formalin (WAKO, Osaka, Japan) for 7–13 days and then sectioned. Representative areas of the brain and spinal cord were embedded in paraffin and cut serially into 6-μm–thick sections. For autopsies conducted prior to April 2011, spinal cords were fixed by using 20 % formalin (226 of 265 subjects with LBAS), whereas spinal cords sampled from April 2011 (39 of 265 subjects with LBAS) and peripheral organs were fixed in 4 % paraformaldehyde in 0.1 M phosphate buffer (pH 7.4) for 48 h. Sections were pretreated with 98 % formic acid for 5 min. For immunohistochemical analysis, we used a phosphorylation dependent anti-α-synuclein monoclonal antibody (pSyn#64; donated by Dr. Iwatsubo, University of Tokyo), which recognizes Ser129. Sections were processed with a Ventana Discovery automatic immunostainer (Roche, Basel, Switzerland). The diameters of the α-synuclein immunoreactive structures were measured visually under a light microscope (Nikon ECLIPSE 80i) with the operator blinded to the clinical and pathological diagnoses.

### Semi-quantitative analysis of LBAS

The degree (density score) of LBAS in the spinal cord, dorsal root, DRG, and sympathetic ganglia was semi-quantitatively graded under a low-power microscopic field (×10) as follows: 0, absent; 1, slight (only thin neurites or small dots less than 12.5 μm in diameter); 2, mild (1–2 intraneuronal cytoplasmic bodies [ICBs] or neurites or dots greater than 12.5 μm in diameter [larger neurites]); 3, moderate (3–5 ICBs or larger neurites); 4, severe (6 or more ICBs or larger neurites). Larger neurites or dots with a diameter greater than 12.5 μm were quantitatively considered to be equivalent to ICBs.

The degree (density score) of LBAS in the brain was based on the third report of the Dementia with Lewy Bodies Consortium [27] as follows: 0, absent; 1, only neurites; 2, 1–3 ICBs; 3, more than 4 ICBs; 4, numerous ICBs filled with extensive immunoreactivity in the neuropil.

### Statistical analysis

Statistical analyzes were performed with the PASW Statistics 18 software (SPSS Japan, Inc.). For multiple comparisons, the Friedman’s chi-square test was used, followed by the Wilcoxon *t*-test with Bonferroni correction as a post-hoc analysis. Correlations between regions were assessed with Spearman’s correlation analysis. The McNemar test was used to test the difference between paired proportions. *P* < 0.05 was considered statistically significant.

## Results

### LBAS in the spinal cord versus in the brain

An additional file shows the clinicopathological characteristics of the 265 subjects who had LBAS in at least one of the sampled anatomical regions [see Additional file 1: Table S1].

When LBAS was present in the spinal cord, all subjects had LBAS in the brain (*n* = 173) (Table 1). No subject had LBAS in the spinal cord without LBAS in the brain (*P* < 0.001).

**Table 1:** Number of subjects with LBAS in the spinal cord or brain, or both

		LBAS in brain	
		(+)	(−)
LBAS in spinal cord	(+)	173	0
	(−)	77	15

*LBAS* Lewy body–related α-synucleinopathy

(+) subjects with LBAS

(−) subjects without LBAS

### LBAS in the spinal cord versus in the brainstem or olfactory bulb plus amygdala

Subjects with LBAS in the brainstem also had LBAS in the spinal cord (87 % [174/200], *P* < 0.001) (Table 2). All subjects with LBAS in the spinal cord also had LBAS in the brainstem. Subjects lacking LBAS in the brainstem invariably failed to show LBAS in the spinal cord (Table 2).

**Table 2:** Number of subjects with LBAS in the spinal cord or brainstem, or both

		LBAS in spinal cord	
		(+)	(−)
LBAS in brainstem	(+)	174	26
	(−)	0	65

*LBAS* Lewy body–related α-synucleinopathy

(+) subjects with LBAS

(−) subjects without LBAS

Subjects with LBAS in the olfactory bulb plus amygdala (*n* = 159) had LBAS in the spinal cord (68.5 % [159/232], *P* = 0.009) (Table 3), and all of those 159 subjects also had LBAS in the brainstem.

**Table 3:** Number of subjects with LBAS in the spinal cord or olfactory bulb plus amygdala

		LBAS in spinal cord	
		(+)	(−)
LBAS in olfactory bulb plus amygdala	(+)	159	73
	(−)	15	18

*LBAS* Lewy body–related α-synucleinopathy

(+) subjects with LBAS

(−) subjects without LBAS

Of the 16 subjects with LBAS in the brainstem but neither in the olfactory bulb nor amygdala, 12 (75 %) had LBAS in the spinal cord. In contrast, subjects with LBAS in the olfactory bulb and amygdala but not in the brainstem (*n* = 51) invariably failed to show LBAS in the spinal cord.

### LBAS density score in the S2 dorsal horn versus in the medullary reticular formation, locus ceruleus, or amygdala

**Fig. 1:**
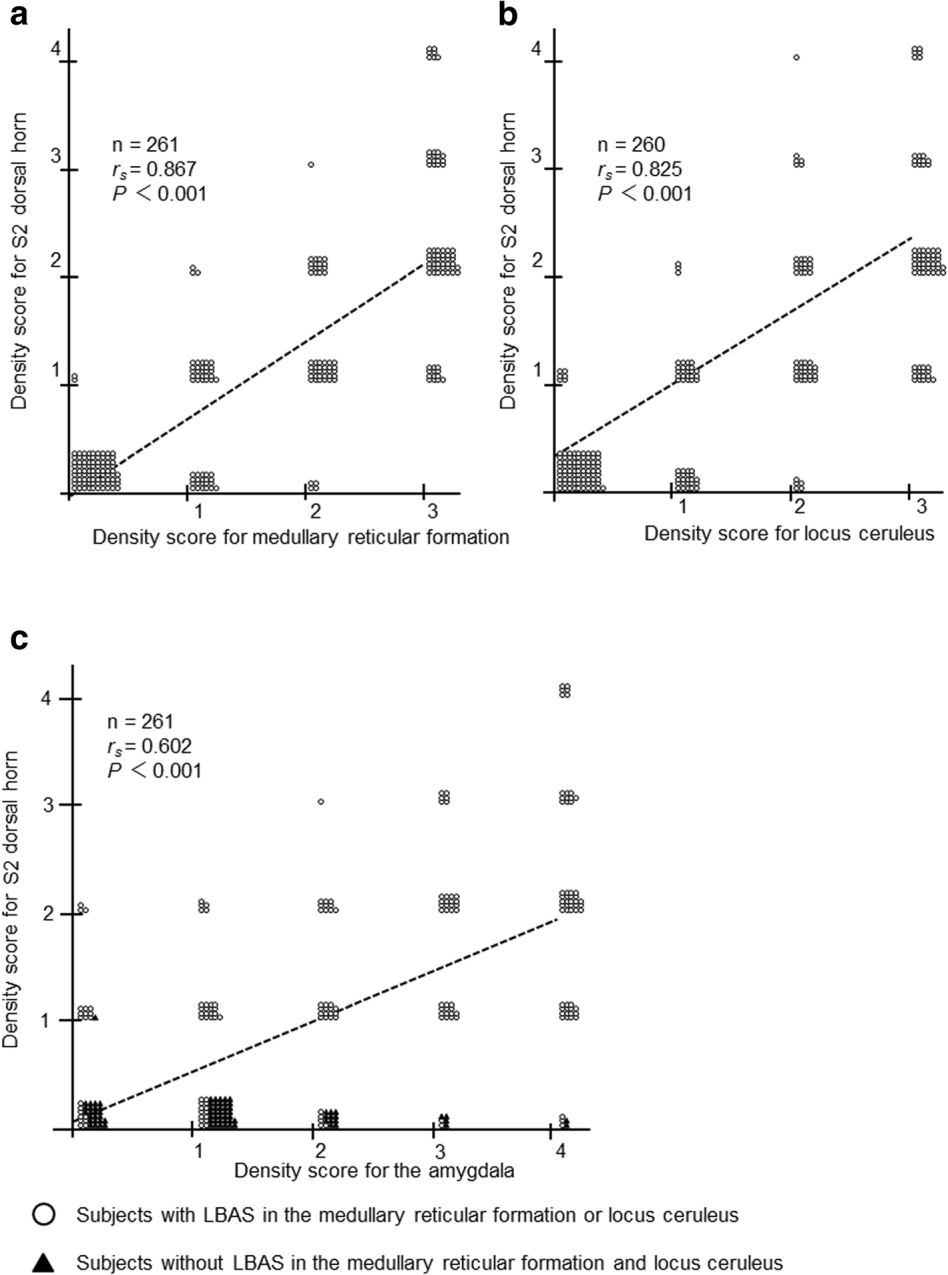
Correlation between LBAS density in specific regions of the brain and in the S2 dorsal horn. Correlation between LBAS density in the medullary reticular formation (**a**), locus ceruleus (**b**), or amygdala (**c**) and in the S2 dorsal horn. Each symbol represents one subject. A density score of 1 or more indicates the deposition of LBAS, with increasing density score indicating greater deposition. Correlation was assessed by calculating Spearman’s rank correlation coefficient. **c** Most subjects with LBAS in the medullary reticular formation or locus ceruleus (open circles) had LBAS in the S2 dorsal horn (S2 dorsal horn density score ≥1). Even when the amygdala showed a higher degree of LBAS (density score 3 or 4), subjects who lacked LBAS in the medullary reticular formation and locus ceruleus (closed triangles) failed to show LBAS in the S2 dorsal horn (S2 dorsal horn density score = 0). LBAS, Lewy body–related α-synucleinopathy

There was a significant correlation (*P* < 0.001) between LBAS density score for the S2 dorsal horn and that for the medullary reticular formation or locus ceruleus in the lower brainstem region (Spearman’s rank correlation coefficient (r_s_), 0.867 and 0.825, respectively; Fig. 1a, b).

Spearman’s rank correlation coefficient of the comparison between the LBAS density scores for the S2 dorsal horn and the amygdala (r_s_ = 0.602) was lower than that for the comparison between the LBAS density scores for the S2 dorsal horn and the medullary reticular formation (r_s_ = 0.867) or locus ceruleus (r_s_ = 0.825) (Fig. 1c). Most subjects with LBAS in the medullary reticular formation or locus ceruleus (Fig. 1c, open circles) had LBAS in the S2 dorsal horn. In contrast, subjects lacking LBAS in the medullary reticular formation and locus ceruleus (Fig. 1c, closed triangles) failed to show LBAS in the S2 dorsal horn.

### Histology of LBAS in the primary sensory neurons

**Fig. 2:**
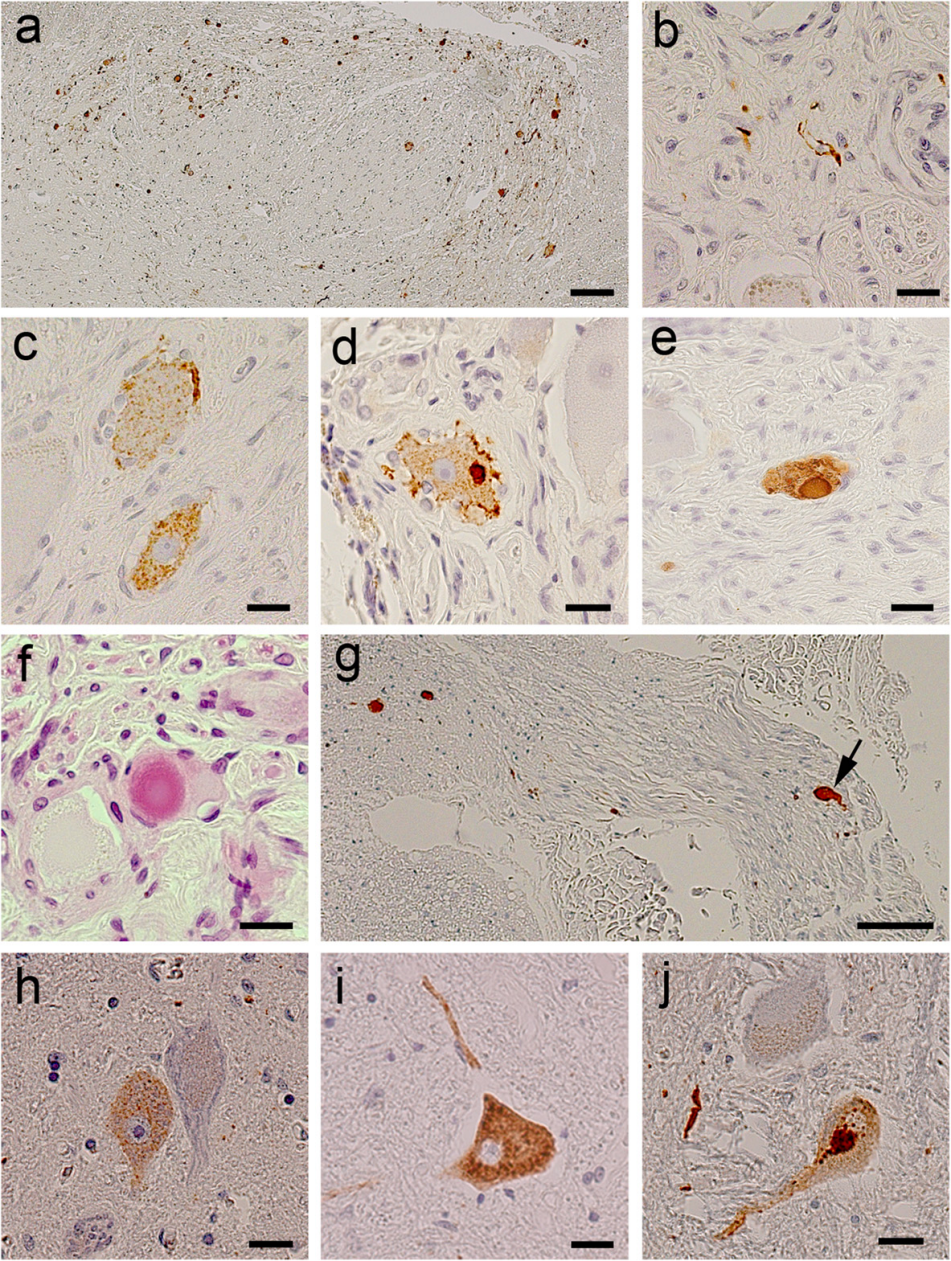
Photomicrographs of paraffin-embedded sections of spinal cord and primary sensory neurons. **a** Sacral dorsal horn of a subject with dementia with Lewy bodies containing numerous LBAS deposits. **b**–**e** Lewy neurites (**b**), neuronal cytoplasmic diffuse granular appearance (**c**), and intraneuronal cytoplasmic bodies (**d**, **e**) in the dorsal root ganglia of subjects with dementia with Lewy bodies. **f** Lewy body in the dorsal root ganglia of a subject with Parkinson’s disease. **g** Proximal portion of the cervical dorsal root showing a swollen neurite (arrow). **h**–**j** LBAS in large motor neurons of the ventral horn of subjects with dementia with Lewy bodies. Neuronal cytoplasmic diffuse granular appearance (**h**, **i**) and intraneuronal cytoplasmic body (**j**). Phosphorylated α-synuclein immunohistochemical staining (**a**–**e**, **g**–**j**) or hematoxylin and eosin (**f**). scale bar = 100 μm for (**a**, **g**); 20 μm for (**b**–**f**, **h**–**j**). LBAS, Lewy body–related α-synucleinopathy

LBAS in the dorsal horn (Fig. 2a) and dorsal root (Fig. 2g) was mainly composed of Lewy neurites. LBAS in the DRG showed Lewy neurites (Fig. 2b), intracytoplasmic, diffuse, granular immunoreactivity (pre-inclusion) (Fig. 2c), ICBs (Fig. 2d, e), or Lewy bodies (Fig. 2f).

### Distribution of LBAS in the primary sensory neurons

Table 4 shows the distribution of LBAS in the primary sensory neurons. Subjects with LBAS in the DRG consistently had LBAS in both the dorsal root and dorsal horn at all lumbosacral levels (e.g., 100 %; 43/43 and 43/43, respectively, in S2). Similarly, all subjects with LBAS in the dorsal root also had LBAS in the dorsal horn (100 %; 90/90, 20/20, 77/77, and 55/55 in S2, S1, L5, and L2, respectively). In contrast, no LBAS was observed in either the DRG or dorsal root when the dorsal horn was not involved (e.g., 0 %; 0/110 and 0/110 in the DRG and dorsal root, respectively, in S2). LBAS was not observed in the DRG when the dorsal root was not involved (e.g., 0 %; 0/171 in S2), except for in one subject (L2; 1/170).

**Table 4:** Distribution of LBAS in primary sensory neurons

		S2 (*n* = 261)			S1 (*n* = 46)			L5 (*n* = 261)			L2 (*n* = 225)		
		DRG	Dorsal	Dorsal	DRG	Dorsal	Dorsal	DRG	Dorsal	Dorsal	DRG	Dorsal	Dorsal
		(+)	root (+)	horn (+)	(+)	root (+)	horn (+)	(+)	root (+)	horn (+)	(+)	root (+)	horn (+)
DRG	(+)	–	43/43	43/43	–	12/12	12/12	–	45/45	45/45	–	34/35	35/35
			(100)	(100)		(100)	(100)		(100)	(100)		(97.1)	(100)
	(−)	–	47/218	108/218	–	8/34	15/34	–	32/216	102/216	–	21/190	83/190
			(21.6)	(49.5)		(23.5)	(44.1)		(14.8)	(47.2)		(11.1)	(43.7)
Dorsal root	(+)	43/90	–	90/90	12/20	–	20/20	45/77	–	77/77	34/55	–	55/55
		(47.8)		(100)	(60)		(100)	(58.4)		(100)	(61.8)		(100)
	(−)	0/171	–	61/171	0/26	–	7/26	0/184	–	70/184	1/170	–	63/170
		(0)		(35.7)	(0)		(26.9)	(0)		(38)	(0.6)		(37.1)
Dorsal horn	(+)	43/151	90/151	–	12/27	20/27	–	45/147	77/147	–	35/118	55/118	–
		(28.5)	(59.6)		(44.4)	(74)		(30.6)	(52.4)		(29.7)	(46.6)	
	(−)	0/110	0/110	–	0/19	0/19	–	0/114	0/114	–	0/107	0/107	–
		(0)	(0)		(0)	(0)		(0)	(0)		(0)	(0)	

Percentage of cases is given in parentheses

*DRG* Dorsal root ganglion, *LBAS* Lewy body-related α-synucleinopathy

(+) subjects with LBAS

(−) subjects without LBAS

### LBAS in the sympathetic ganglia and Th-IML

**Fig. 3:**
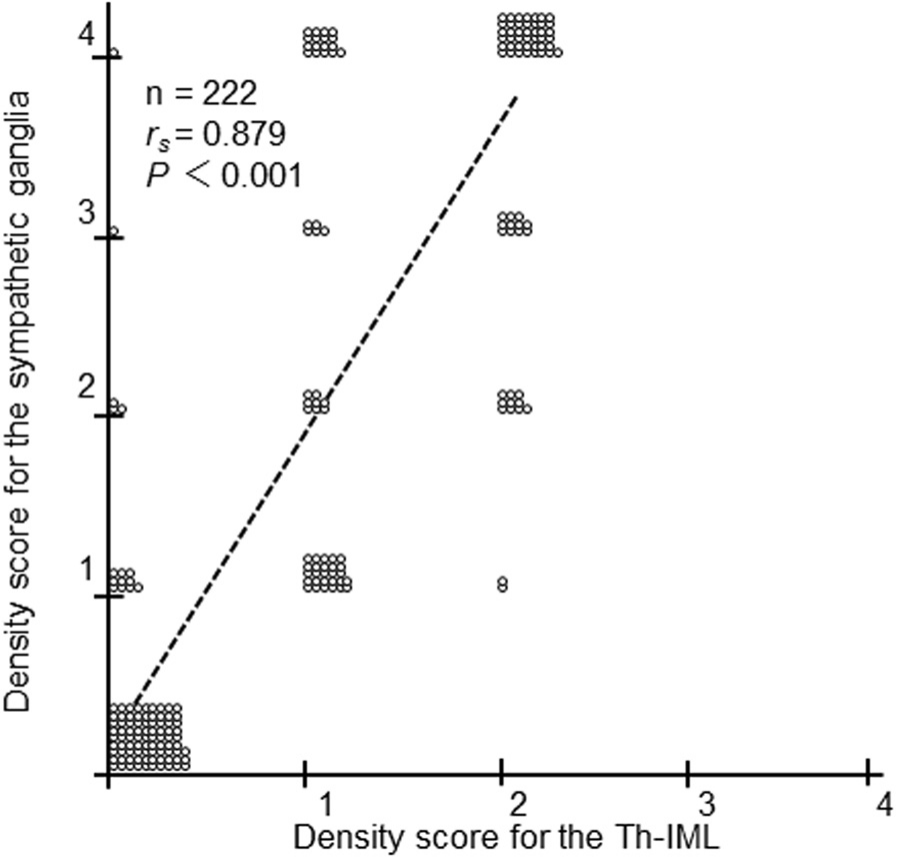
Correlation between the LBAS density for the Th-IML and that for the sympathetic ganglia. Each symbol represents one subject. A density score of 1 or more indicates the deposition of LBAS, with increasing density score indicating greater deposition. Subjects who had LBAS in the Th-IML (density score for the Th-IML ≥1) invariably showed LBAS in the sympathetic ganglia (density score for the sympathetic ganglia ≥1). Correlation was assessed by calculating Spearman’s rank correlation coefficient. LBAS, Lewy body–related α-synucleinopathy

There was a strong correlation between LBAS density score in the sympathetic ganglia and that in the Th-IML (*r*_*s*_ = 0.879, *P* < 0.001, Fig. 3). When LBAS was present in the Th-IML, all subjects had LBAS in the sympathetic ganglia (Fig. 3).

Of 222 subjects whom both the sympathetic ganglia and Th-IML were sampled, 12 (5.4 %) had LBAS in the sympathetic ganglia, even in the absence of LBAS in the whole brain. Of these 12 subjects, three had LBAS in the Th-IML. Of these three subjects, one had LBAS only in the Th-IML and not in any other region of the spinal cord.

### Intersegmental comparison of LBAS density in the spinal cord

**Fig. 4:**
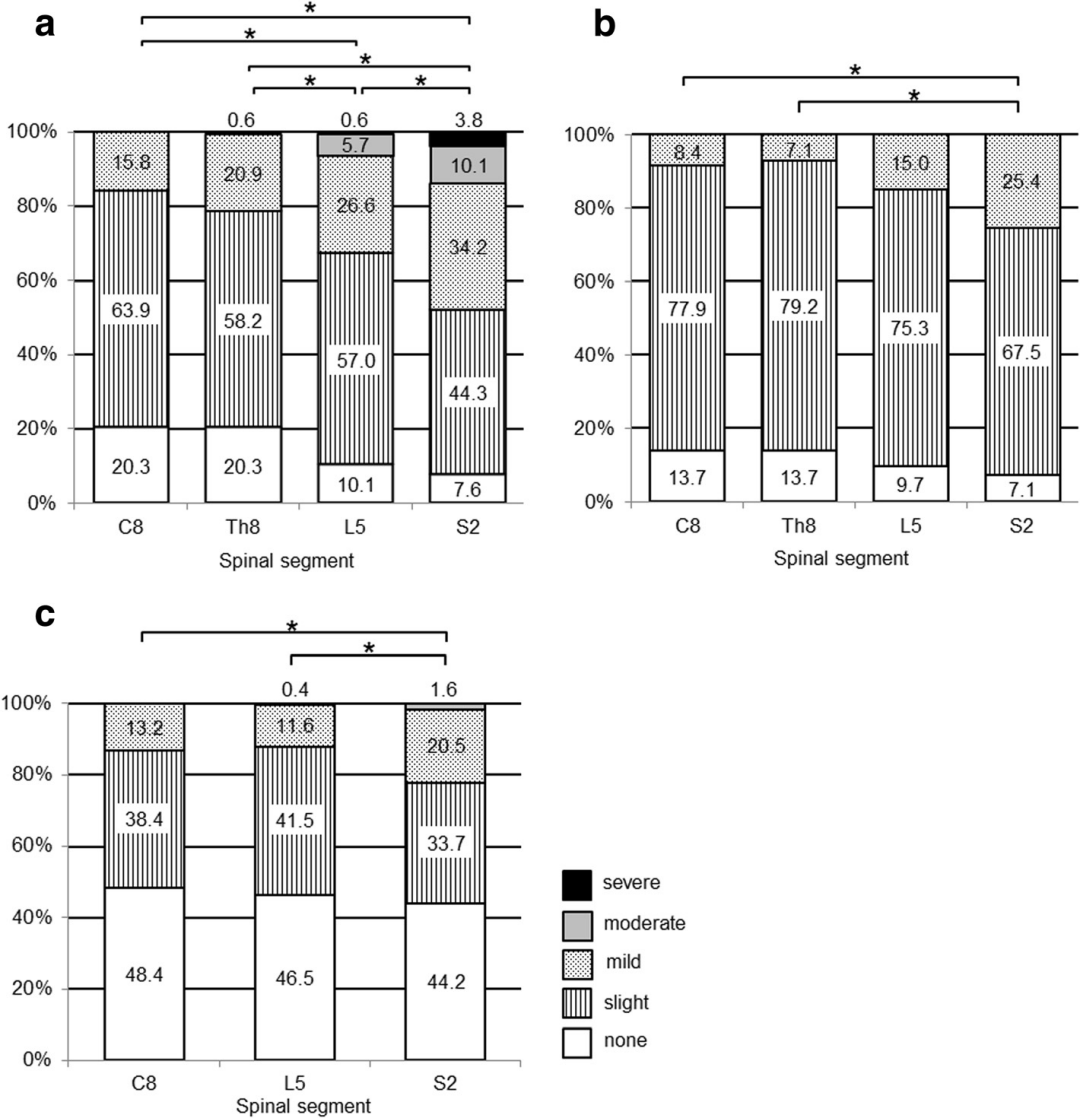
Comparison of LBAS density at different levels of the spinal cord. The degree of LBAS among four different segments (cervical, thoracic, lumbar, and sacral) in three different regions (dorsal horn, ventral horn, and intermediate zone) of the spinal gray matter showed a caudo–rostral gradient in which the greatest was at the sacral level followed by the lumbar, thoracic, and cervical levels. **a** Dorsal horn (Rexed lamina I–VI, *n* = 256). **b** Ventral horn (Rexed lamina VIII–IX, *n* = 258). **c** Intermediate zone (Rexed lamina VII and X, *n* = 258). * *P* < 0.01

Comparing the density score of LBAS in the dorsal horn among four segments (cervical, thoracic, lumbar, and sacral) of the spinal cord, we found a caudo–rostral gradient in which the greatest LBAS density score was at the sacral level followed by the lumbar, thoracic, and cervical levels (Fig. 4a). Similar results were obtained in the ventral horn (Fig. 4b) and intermediate zone (Fig. 4c).

### LBAS in large motor neurons

In the ventral horn, not only Lewy neurites, but also LBAS in the cell soma of the large motor neurons was observed (Fig. 2h–j). In symptomatic subjects (Parkinson’s disease and dementia with Lewy bodies) (*n* = 55) only, the frequency of ICBs in the large motor neurons was 27.3, 21.8, 23.6 and 63.6 % in C8, Th8, L5, and S2, respectively (Additional file 2: Figure S1).

### LBAS in the Sa-IML

Of 261 subjects examined, no subject had LBAS that was limited to the Sa-IML. Those with LBAS in the Sa-IML also had LBAS in other sites of the spinal cord.

## Discussion

In the present study, we examined the distribution of LBAS in the spinal cord, primary sensory neurons, and preganglionic sympathetic nerves in a consecutive series of elderly autopsy subjects. Our results suggest that 1) the presence of LBAS in specific regions of the lower brainstem (i.e., the medullary reticular formation and locus ceruleus) is strongly correlated with the presence of LBAS in the spinal cord, whereas the presence of LBAS in the spinal cord is independent of the presence of LBAS in the olfactory bulb and amygdala; 2) LBAS propagates retrogradely from the dorsal horn to the DRG through the dorsal root; 3) LBAS propagates retrogradely from the sympathetic ganglia to the Th-IML; and 4) LBAS in the spinal cord predominantly accumulates at the caudal level.

### Association between LBAS in the spinal cord and that in the brainstem or amygdala

**Fig. 5:**
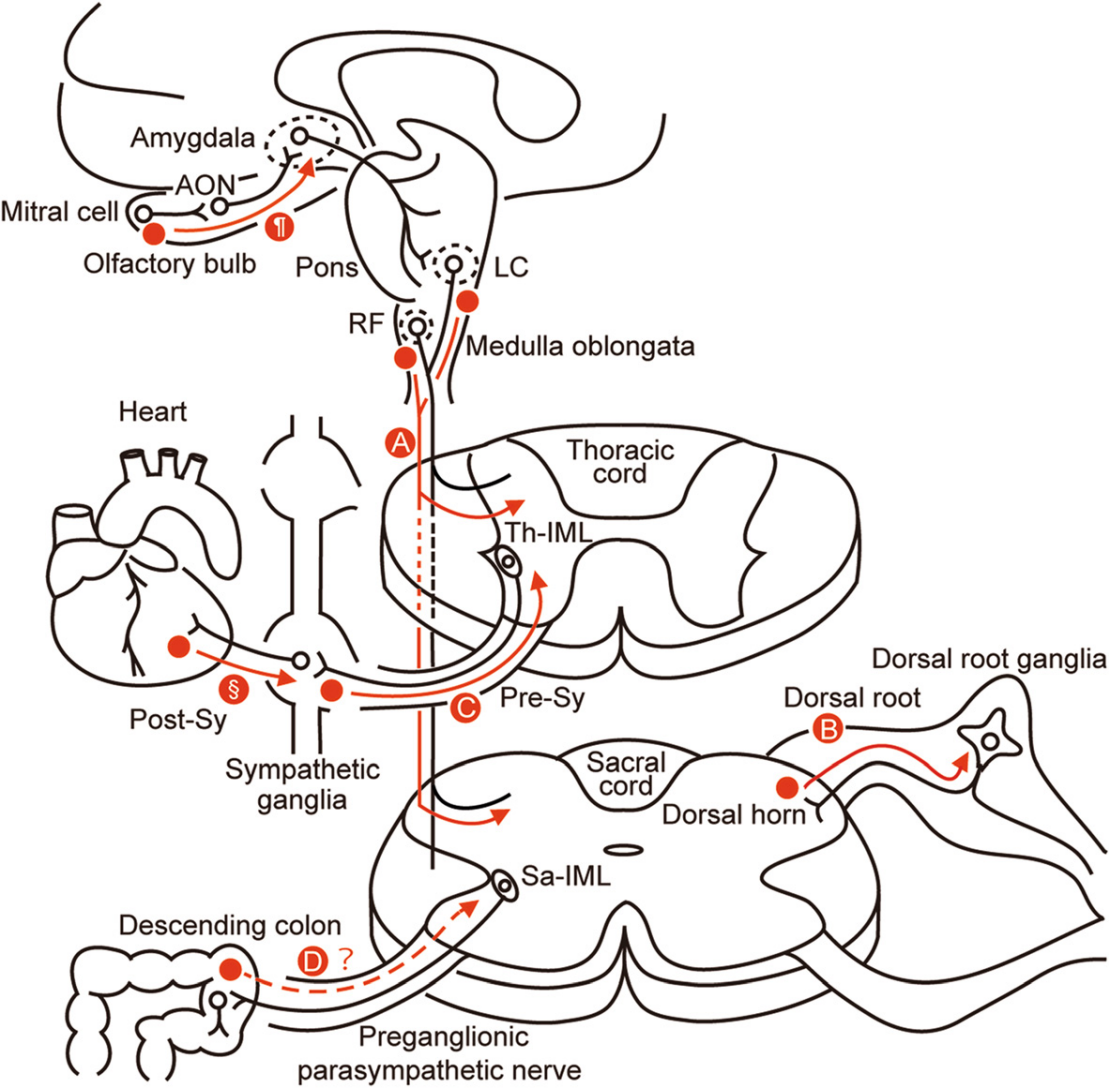
Schematic summarizing the propagation of α-synuclein. Red circles represent pathologic α-synuclein accumulation. Proposed pathway of the spread of LBAS from the RF or LC to the spinal gray matter (A), in the primary sensory neurons (B), in the preganglionic sympathetic nerves (C), and in the preganglionic parasympathetic nerves (D). AON, anterior olfactory nucleus; LC, locus ceruleus; Post-Sy, postganglionic sympathetic nerve; Pre-Sy, preganglionic sympathetic nerve; RF, medullary reticular formation; Sa-IML, nucleus of intermediolateral column of the sacral cord; Th-IML, nucleus of intermediolateral column of the thoracic cord. Pathway of the spread of LBAS in the olfactory nervous system (¶) [16, 18], and in cardiac postganglionic sympathetic nerves (§) [2]

Here we found that LBAS develops first in the brain before developing in the spinal cord, which is consistent with the findings of a previous study [20]. We found that LBAS in the spinal cord was associated with LBAS in the medullary reticular formation and locus ceruleus. Previous studies have revealed the existence of direct connectivity via projecting neurons between the spinal cord and the medullary reticular formation and locus ceruleus, but not the amygdala [23–26, 33, 34]. Our findings support the hypothesis of Del Tredici and Braak in which LBAS in the spinal cord descends from the supraspinal medullary reticular formation, locus ceruleus, and raphe nucleus via projecting neurons (Fig. 5a) [20]. In addition, we revealed a novel finding that LBAS in the spinal cord is not correlated with LBAS in the olfactory bulb and amygdala. When LBAS follows the olfactory bulb–amygdala pathway and develops downward through the spinal cord, the lower brainstem is likely a key structure, implying that in Lewy body disease that is following the olfactory bulb–amygdala pathway, LBAS primarily develops in the amygdala and then gradually spreads to the lower brainstem and finally the spinal cord.

### LBAS in primary sensory neurons and preganglionic sympathetic nerves

The present study is the first to evaluate how LBAS spreads in primary sensory neurons. The dorsal root is composed of axons derived from the DRG. LBAS primarily develops in the dorsal horn and then spreads to the DRG though the dorsal root (i.e., central-to-peripheral spreading) (Fig. 5b).

Regarding the preganglionic sympathetic nerves, the sympathetic ganglia may be one of the starting points for LBAS development, and LBAS may propagate from the sympathetic ganglia to the Th-IML (i.e., peripheral-to-central spreading) (Fig. 5c).

Our results suggest that LBAS spreads retrogradely through the primary sensory neurons and preganglionic sympathetic nerves, which is plausible because the axonal development of LBAS precedes perikaryal aggregation [35–39]. Indeed, LBAS has already been shown to spread retrogradely in cardiac postganglionic sympathetic nerves (Fig. 5§) [2]. Recent *in vivo* studies have revealed that LBAS also spreads retrogradely along the vagus nerve from the gut [40], and along the dopaminergic neurons in the nigrostriatal system [41]. In the olfactory nervous system, LBAS spreads both anterogradely (Fig. 5¶) and retrogradely from the periphery of the olfactory bulb to interconnected regions [16, 18, 33, 42]. Whether retrograde spread alone explains the Lewy body disease process needs to be further examined.

### Caudo–rostral gradient in the spinal cord

The amount of LBAS in the spinal gray matter showed a caudo–rostral gradient, which is consistent with previous descriptions [3, 20, 22]. The precise reason for this gradient is unclear, but may well be ascribed to 1) a selective caudo–rostral vulnerability to pathologic α-synuclein accumulation, or 2) the sacral segment having a higher amount of endogenous α-synuclein. Lewy neurites may play a direct role in peripheral nerve damage [43–45]. Parkinson’s disease patients often suffer from limb pain, especially in the lower limbs, which is localized to the anatomical territory of a peripheral nerve or nerve root [46–49]. The development of LBAS in primary sensory neurons and the development of the caudo-rostral gradient may be associated with sensory disorders in Parkinson’s disease patients.

### LBAS in large motor neurons

LBAS in the large motor neurons preferentially accumulated at the lower level of the spinal cord. Recent studies indicate the subclinical denervation especially in the lower limbs in Parkinson’s disease [50, 51]. It remains unclear whether LBAS propagates anterogradely or retrogradely in large motor neurons. One experimental study has shown retrograde spread of LBAS into the spinal cord via the peripheral nerves in transgenic mice who received intramuscular injection of pathogenic α-synuclein [52].

### LBAS in Sa-IML

Given the early development of gastrointestinal abnormalities in Lewy body disease [53–56], we hypothesized that the Sa-IML would be one of the initial sites where LBAS develops through the preganglionic parasympathetic nerves from pelvic organs such as the descending colon or rectum (Fig. 5d) [15, 57]. However, we did not find any subject harboring LBAS exclusively in the Sa-IML. One reason for this may be that the S2 level we examined contains fewer neurons than the S3–5 levels, which reduced the potential for detecting LBAS in the Sa-IML. Further, investigation of both the pelvic organs and Sa-IML will be important to clarify whether an alternative pattern of LBAS propagation, in which LBAS originates in the Sa-IML before spreading to the entire spinal cord, exists or not.

### Future direction

The findings of the present neuropathological examination should be interpreted with caution because these results do not mean that LBAS in the spinal cord always originates from outside the spinal cord. Some LBAS in the spinal cord may be derived from single or multiple sites within the spinal cord. Furthermore, the study cohort included only elderly subjects and therefore the results may not be generalizable to younger populations. Our neuropathological assessment did not allow us to see the actual process of LBAS propagation within a neuron; therefore, clinicopathological studies are required to establish a causal relationship between LBAS and peripheral nerve disorders in Lewy body diseases.

## Conclusion

LBAS in the spinal cord was associated with the lower regions of the brainstem but was not associated with the olfactory bulb and amygdala. LBAS may spread centrifugally along the primary sensory neurons, whereas it may spread centripetally along the preganglionic sympathetic nerves. Further study of the progressive spread of LBAS in connected neuronal systems will assist in clarifying the precise progression of the Lewy body diseases.

## Availability of supporting data

The data sets supporting the results of this article are included within the article and its additional files.

## Additional files

Additional file 1: Table S1. Clinicopathological characteristics of 265 subjects with LBAS. (XLSX 12 kb) Additional file 2: Figure S1. Intraneuronal cytoplasmic bodies in the large motor neurons. (PDF 93 kb)

## References

[1] Spillantini MG, Schmidt ML, Lee VM, Trojanowski JQ, Jakes R, Goedert M (1997). Alpha-synuclein in Lewy bodies. Nature.

[2] Orimo S, Uchihara T, Nakamura A, Mori F, Kakita A, Wakabayashi K, Takahashi H (2008). Axonal alpha-synuclein aggregates herald centripetal degeneration of cardiac sympathetic nerve in Parkinson’s disease. Brain.

[3] Beach TG, Adler CH, Sue LI, Vedders L, Lue L, White Iii CL, Akiyama H, Caviness JN, Shill HA, Sabbagh MN, Walker DG, Arizona Parkinson’s Disease C (2010). Multi-organ distribution of phosphorylated alpha-synuclein histopathology in subjects with Lewy body disorders. Acta Neuropathol.

[4] Wakabayashi K, Mori F, Tanji K, Orimo S, Takahashi H (2010). Involvement of the peripheral nervous system in synucleinopathies, tauopathies and other neurodegenerative proteinopathies of the brain. Acta Neuropathol.

[5] Kordower JH, Chu Y, Hauser RA, Freeman TB, Olanow CW (2008). Lewy body-like pathology in long-term embryonic nigral transplants in Parkinson’s disease. Nat Med.

[6] Li JY, Englund E, Holton JL, Soulet D, Hagell P, Lees AJ, Lashley T, Quinn NP, Rehncrona S, Bjorklund A, Widner H, Revesz T, Lindvall O, Brundin P (2008). Lewy bodies in grafted neurons in subjects with Parkinson’s disease suggest host-to-graft disease propagation. Nat Med.

[7] Masuda-Suzukake M, Nonaka T, Hosokawa M, Oikawa T, Arai T, Akiyama H, Mann DM, Hasegawa M (2013). Prion-like spreading of pathological alpha-synuclein in brain. Brain.

[8] Brundin P, Li JY, Holton JL, Lindvall O, Revesz T (2008). Research in motion: the enigma of Parkinson’s disease pathology spread. Nat Rev Neurosci.

[9] Goedert M, Clavaguera F, Tolnay M (2010). The propagation of prion-like protein inclusions in neurodegenerative diseases. Trends Neurosci.

[10] Luk KC, Kehm VM, Zhang B, O’Brien P, Trojanowski JQ, Lee VM (2012). Intracerebral inoculation of pathological alpha-synuclein initiates a rapidly progressive neurodegenerative alpha-synucleinopathy in mice. J Exp Med.

[11] Mougenot AL, Nicot S, Bencsik A, Morignat E, Verchere J, Lakhdar L, Legastelois S, Baron T (2012). Prion-like acceleration of a synucleinopathy in a transgenic mouse model. Neurobiol Aging.

[12] Olanow CW, Prusiner SB (2009). Is Parkinson’s disease a prion disorder?. Proc Natl Acad Sci U S A.

[13] Luk KC, Kehm V, Carroll J, Zhang B, O’Brien P, Trojanowski JQ, Lee VM (2012). Pathological alpha-synuclein transmission initiates Parkinson-like neurodegeneration in nontransgenic mice. Science.

[14] Desplats P, Lee HJ, Bae EJ, Patrick C, Rockenstein E, Crews L, Spencer B, Masliah E, Lee SJ (2009). Inclusion formation and neuronal cell death through neuron-to-neuron transmission of alpha-synuclein. Proc Natl Acad Sci U S A.

[15] Pan-Montojo F, Anichtchik O, Dening Y, Knels L, Pursche S, Jung R, Jackson S, Gille G, Spillantini MG, Reichmann H, Funk RH (2010). Progression of Parkinson’s disease pathology is reproduced by intragastric administration of rotenone in mice. PLoS One.

[16] Hawkes CH, Del Tredici K, Braak H (2007). Parkinson’s disease: a dual-hit hypothesis. Neuropathol Appl Neurobiol.

[17] Braak H, Del Tredici K, Rub U, de Vos RA, Jansen Steur EN, Braak E (2003). Staging of brain pathology related to sporadic Parkinson’s disease. Neurobiol Aging.

[18] Sengoku R, Saito Y, Ikemura M, Hatsuta H, Sakiyama Y, Kanemaru K, Arai T, Sawabe M, Tanaka N, Mochizuki H, Inoue K, Murayama S (2008). Incidence and extent of Lewy body-related alpha-synucleinopathy in aging human olfactory bulb. J Neuropathol Exp Neurol.

[19] Zaccai J, Brayne C, McKeith I, Matthews F, Ince PG (2008). Patterns and stages of alpha-synucleinopathy: relevance in a population-based cohort. Neurology.

[20] Del Tredici K, Braak H (2012). Spinal cord lesions in sporadic Parkinson’s disease. Acta Neuropathol.

[21] Braak H, Sastre M, Bohl JR, de Vos RA, Del Tredici K (2007). Parkinson’s disease: lesions in dorsal horn layer I, involvement of parasympathetic and sympathetic pre- and postganglionic neurons. Acta Neuropathol.

[22] Tamura T, Yoshida M, Hashizume Y, Sobue G (2012). Lewy body-related alpha-synucleinopathy in the spinal cord of cases with incidental Lewy body disease. Neuropathology.

[23] Bowsher D (1957). Termination of the central pain pathway in man: the conscious appreciation of pain. Brain.

[24] Kojima M, Sano Y (1983). The organization of serotonin fibers in the anterior column of the mammalian spinal cord. An immunohistochemical study. Anat Embryol (Berl).

[25] Westlund KN, Bowker RM, Ziegler MG, Coulter JD (1982). Descending noradrenergic projections and their spinal terminations. Prog Brain Res.

[26] Sah P, Faber ES, Lopez De Armentia M, Power J (2003). The amygdaloid complex: anatomy and physiology. Physiol Rev.

[27] McKeith IG, Dickson DW, Lowe J, Emre M, O’Brien JT, Feldman H, Cummings J, Duda JE, Lippa C, Perry EK, Aarsland D, Arai H, Ballard CG, Boeve B, Burn DJ, Costa D, Del Ser T, Dubois B, Galasko D, Gauthier S, Goetz CG, Gomez-Tortosa E, Halliday G, Hansen LA, Hardy J, Iwatsubo T, Kalaria RN, Kaufer D, Kenny RA, Korczyn A, Kosaka K, Lee VM, Lees A, Litvan I, Londos E, Lopez OL, Minoshima S, Mizuno Y, Molina JA, Mukaetova-Ladinska EB, Pasquier F, Perry RH, Schulz JB, Trojanowski JQ, Yamada M (2005). Diagnosis and management of dementia with Lewy bodies: third report of the DLB Consortium. Neurology.

[28] Saito Y, Ruberu NN, Sawabe M, Arai T, Kazama H, Hosoi T, Yamanouchi H, Murayama S (2004). Lewy body-related alpha-synucleinopathy in aging. J Neuropathol Exp Neurol.

[29] Braak H, Braak E (1991). Neuropathological stageing of Alzheimer-related changes. Acta Neuropathol.

[30] Mirra SS, Heyman A, McKeel D, Sumi SM, Crain BJ, Brownlee LM, Vogel FS, Hughes JP, van Belle G, Berg L (1991). The Consortium to Establish a Registry for Alzheimer’s Disease (CERAD). Part II. Standardization of the neuropathologic assessment of Alzheimer’s disease. Neurology.

[31] Thal DR, Rub U, Orantes M, Braak H (2002). Phases of A beta-deposition in the human brain and its relevance for the development of AD. Neurology.

[32] Saito Y, Ruberu NN, Sawabe M, Arai T, Tanaka N, Kakuta Y, Yamanouchi H, Murayama S (2004). Staging of argyrophilic grains: an age-associated tauopathy. J Neuropathol Exp Neurol.

[33] Lerner A, Bagic A (2008). Olfactory pathogenesis of idiopathic Parkinson disease revisited. Mov Disord.

[34] Zemlan FP, Behbehani MM, Beckstead RM (1984). Ascending and descending projections from nucleus reticularis magnocellularis and nucleus reticularis gigantocellularis: an autoradiographic and horseradish peroxidase study in the rat. Brain Res.

[35] Marui W, Iseki E, Nakai T, Miura S, Kato M, Ueda K, Kosaka K (2002). Progression and staging of Lewy pathology in brains from patients with dementia with Lewy bodies. J Neurol Sci.

[36] Wakabayashi K, Hayashi S, Kakita A, Yamada M, Toyoshima Y, Yoshimoto M, Takahashi H (1998). Accumulation of alpha-synuclein/NACP is a cytopathological feature common to Lewy body disease and multiple system atrophy. Acta Neuropathol.

[37] Wakabayashi K, Tanji K, Mori F, Takahashi H (2007). The Lewy body in Parkinson’s disease: Molecules implicated in the formation and degradation of α-synuclein aggregates. Neuropathology.

[38] Saito Y, Kawashima A, Ruberu NN, Fujiwara H, Koyama S, Sawabe M, Arai T, Nagura H, Yamanouchi H, Hasegawa M, Iwatsubo T, Murayama S (2003). Accumulation of phosphorylated alpha-synuclein in aging human brain. J Neuropathol Exp Neurol.

[39] Braak H, Sandmann-Keil D, Gai W, Braak E (1999). Extensive axonal Lewy neurites in Parkinson’s disease: a novel pathological feature revealed by alpha-synuclein immunocytochemistry. Neurosci Lett.

[40] Holmqvist S, Chutna O, Bousset L, Aldrin-Kirk P, Li W, Bjorklund T, Wang ZY, Roybon L, Melki R, Li JY (2014). Direct evidence of Parkinson pathology spread from the gastrointestinal tract to the brain in rats. Acta Neuropathol.

[41] Recasens A, Dehay B, Bove J, Carballo-Carbajal I, Dovero S, Perez-Villalba A, Fernagut PO, Blesa J, Parent A, Perier C, Farinas I, Obeso JA, Bezard E, Vila M (2014). Lewy body extracts from Parkinson disease brains trigger alpha-synuclein pathology and neurodegeneration in mice and monkeys. Ann Neurol.

[42] Rey NL, Petit GH, Bousset L, Melki R, Brundin P (2013). Transfer of human alpha-synuclein from the olfactory bulb to interconnected brain regions in mice. Acta Neuropathol.

[43] Donadio V, Incensi A, Leta V, Giannoccaro MP, Scaglione C, Martinelli P, Capellari S, Avoni P, Baruzzi A, Liguori R (2014). Skin nerve alpha-synuclein deposits: a biomarker for idiopathic Parkinson disease. Neurology.

[44] Nolano M, Provitera V, Estraneo A, Selim MM, Caporaso G, Stancanelli A, Saltalamacchia AM, Lanzillo B, Santoro L (2008). Sensory deficit in Parkinson’s disease: evidence of a cutaneous denervation. Brain.

[45] Doppler K, Ebert S, Uceyler N, Trenkwalder C, Ebentheuer J, Volkmann J, Sommer C (2014). Cutaneous neuropathy in Parkinson’s disease: a window into brain pathology. Acta Neuropathol.

[46] Defazio G, Berardelli A, Fabbrini G, Martino D, Fincati E, Fiaschi A, Moretto G, Abbruzzese G, Marchese R, Bonuccelli U, Del Dotto P, Barone P, De Vivo E, Albanese A, Antonini A, Canesi M, Lopiano L, Zibetti M, Nappi G, Martignoni E, Lamberti P, Tinazzi M (2008). Pain as a nonmotor symptom of Parkinson disease: evidence from a case–control study. Arch Neurol.

[47] Beiske AG, Loge JH, Ronningen A, Svensson E (2009). Pain in Parkinson’s disease: prevalence and characteristics. Pain.

[48] Goetz CG, Tanner CM, Levy M, Wilson RS, Garron DC (1986). Pain in Parkinson’s disease. Mov Disord.

[49] Negre-Pages L, Regragui W, Bouhassira D, Grandjean H, Rascol O, DoPaMi PSG (2008). Chronic pain in Parkinson’s disease: the cross-sectional French DoPaMiP survey. Mov Disord.

[50] Caviness JN, Smith BE, Stevens JC, Adler CH, Caselli RJ, Reiners CA, Hentz JG, Muenter MD (2000). Motor unit changes in sporadic idiopathic Parkinson’s disease. Mov Disord.

[51] Caviness JN, Smith BE, Clarke Stevens J, Adler CH, Caselli RJ, Hentz JG, Manfred MS, Muenter D (2002). Motor unit number estimates in idiopathic Parkinson’s disease. Parkinsonism Relat Disord.

[52] Sacino AN, Brooks M, Thomas MA, McKinney AB, Lee S, Regenhardt RW, McGarvey NH, Ayers JI, Notterpek L, Borchelt DR, Golde TE, Giasson BI (2014). Intramuscular injection of alpha-synuclein induces CNS alpha-synuclein pathology and a rapid-onset motor phenotype in transgenic mice. Proc Natl Acad Sci U S A.

[53] Lebouvier T, Chaumette T, Damier P, Coron E, Touchefeu Y, Vrignaud S, Naveilhan P, Galmiche JP, Bruley des Varannes S, Derkinderen P, Neunlist M (2008). Pathological lesions in colonic biopsies during Parkinson’s disease. Gut.

[54] Kuo YM, Li Z, Jiao Y, Gaborit N, Pani AK, Orrison BM, Bruneau BG, Giasson BI, Smeyne RJ, Gershon MD, Nussbaum RL (2010). Extensive enteric nervous system abnormalities in mice transgenic for artificial chromosomes containing Parkinson disease-associated alpha-synuclein gene mutations precede central nervous system changes. Hum Mol Genet.

[55] Abbott RD, Petrovitch H, White LR, Masaki KH, Tanner CM, Curb JD, Grandinetti A, Blanchette PL, Popper JS, Ross GW (2001). Frequency of bowel movements and the future risk of Parkinson’s disease. Neurology.

[56] Braak H, de Vos RA, Bohl J, Del Tredici K (2006). Gastric alpha-synuclein immunoreactive inclusions in Meissner’s and Auerbach’s plexuses in cases staged for Parkinson’s disease-related brain pathology. Neurosci Lett.

[57] Pan-Montojo F, Schwarz M, Winkler C, Arnhold M, O’Sullivan GA, Pal A, Said J, Marsico G, Verbavatz JM, Rodrigo-Angulo M, Gille G, Funk RH, Reichmann H (2012). Environmental toxins trigger PD-like progression via increased alpha-synuclein release from enteric neurons in mice. Sci Rep.

