# Supplementary material for: Distribution of α-synuclein in the spinal cord and dorsal root ganglia in an autopsy cohort of elderly persons
Source: Acta Neuropathol Commun. 2015 Sep 15;3:57. doi: 10.1186/s40478-015-0236-9 (PMC4571135; doi:10.1186/s40478-015-0236-9)
Supplement: Additional file 2: Figure S1. — Intraneuronal cytoplasmic bodies in the large motor neurons. (PDF 93 kb) [file 40478_2015_236_MOESM2_ESM.pdf]

Additional file 2: Figure S1

Frequency of intraneuronal cytoplasmic bodies (ICBs) in the large motor neurons at different levels of the ventral horn in symptomatic subjects

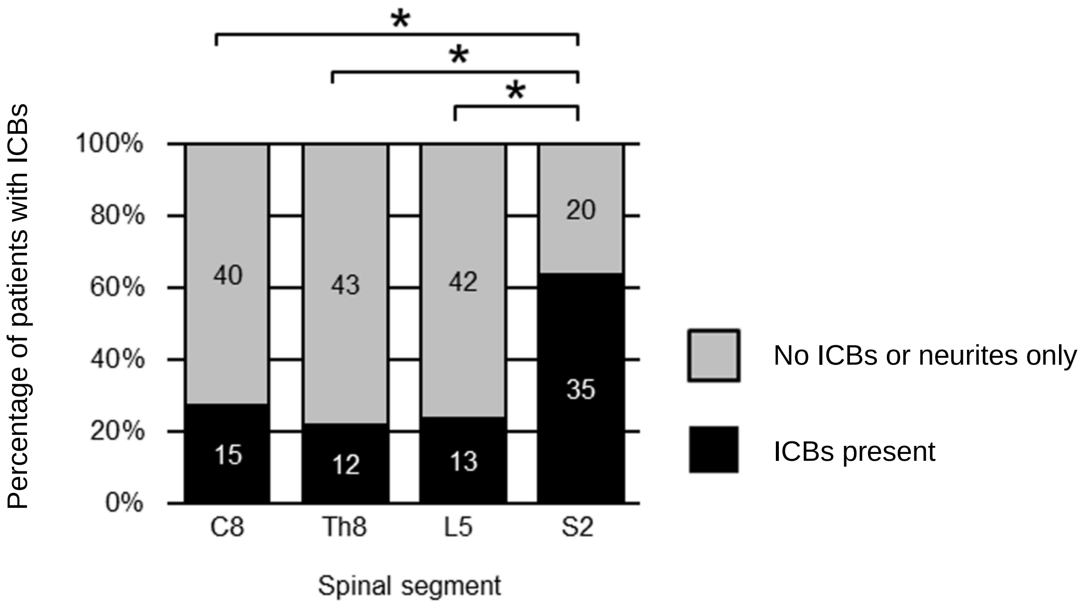

Symptomatic subjects (Parkinson's disease and dementia with Lewy bodies) (n = 55). *P < 0.01. ICBs, intraneuronal cytoplasmic bodies.
